# The Relationship Between Bone Mineral Density and Lumbar Disc Herniation in Postmenopausal Women

**DOI:** 10.7759/cureus.44156

**Published:** 2023-08-26

**Authors:** Zekeriya Ersin Çelen, Tolga Onay

**Affiliations:** 1 Department of Orthopaedic Surgery and Traumatology, Yalova Training and Research Hospital, Yalova, TUR; 2 Department of Orthopaedic Surgery and Traumatology, Göztepe Training and Research Hospital, İstanbul, TUR

**Keywords:** cross sectional study, postmenopausal women, low back pain, disc herniation, bone mineral density

## Abstract

Introduction: In previous studies, the relationship between BMD (bone mineral density) and LDH (lumbar disc herniation) has been investigated in young people, except for postmenopausal women. The aim of this study was to evaluate this association in postmenopausal women.

Methods: A cross-sectional analysis of 545 consecutive postmenopausal women was performed at a single center. The study included patients aged 45 to 85 with low back pain. Age, weight, height, L1-L4 BMD, L1-L4 T-score, L1-L4 Z-score, femoral neck BMD, femoral neck T-score, and femoral neck Z-score of patients were collected. MRI scans were assessed for the diagnosis of LDH. To explore the impact of the number of herniated segments, patients with LDH were further divided into single-level and multi-level LDH groups.

Results: Five hundred and thirteen postmenopausal women were included in the final analysis. The mean age of the patients was 61.3±8.6 years in the LDH group and 58.4±7.8 years in the non-LDH group (p=0.001). The LDH group had higher lumbar (p<0.001) and femoral neck (p<0.05) BMD, T, and Z-scores than the non-LDH group. In binary logistic regression analysis, age, lumbar, and femoral neck BMD were significantly associated with LDH (p<0.001, p=0.03, and p=0.040, respectively). Patients with multi-level herniation had significantly higher rates of obesity (BMI ≥30) compared to patients with single-level herniation (58.0% vs. 47.0%; p=0.031). However, in terms of obesity rates, the LDH group and the non-LDH group were statistically similar (53.9% vs. 54.2%; p=0.961). There was no association between the single and multi-level herniation groups in terms of L1-4 and femoral neck BMD (p=0.760 and 0.435, respectively).

Conclusion: Higher lumbar bone mineral density and higher femoral neck bone mineral densities were found to be associated with lumber disc herniation in postmenopausal women with low back pain. These results suggest that bone mineral density assessment may be useful in clinical practice to determine which patients are at higher risk of lumbar disc herniation.

## Introduction

Lumbar disc herniation (LDH) is one of the most frequent diseases that has a serious impact on people's quality of life and puts a heavy burden on families and society [1]. It is also the most frequent cause of sciatica and affects 1% to 5% of the population every year [2,3]. Therefore, it is important to elucidate the risk factors and the pathophysiological mechanisms involved in the development of LDH. Clarifying these mechanisms may enable prevention or early diagnosis of the disease. Previously reported associated factors include smoking, genetic factors, occupational factors, and obesity [4-6]. In addition, a recent study reported that symptomatic LDH increases with age, and the incidence is higher in women than in men [7].

Since bone mineral density (BMD) levels affect not only the structure of bones but also nonosseous tissues, osteopenia and osteoporosis disrupt the morphology of lumbar vertebral bones and intervertebral discs [8,9]. On the other hand, it has also been shown that stronger bones may be associated with more disc degeneration [9]. Nevertheless, although lumbar vertebral bones and lumbar intervertebral discs are close to each other both biomechanically and anatomically, to our knowledge, the association between BMD and LDH has not been studied in postmenopausal women before. Two studies were detected evaluating this relationship in young healthy people living in an urban area and young patients, with the exclusion of postmenopausal women, and the studies found no significant relationship [10,11].

Close contact between the vertebral bone marrow and the vertebral endplate is important for adequate nutrition of the nucleus pulposus of the disc [12]. High BMD causes increased end-plate resistance and affects the vascularization of the disc by disrupting diffusion [13]. Furthermore, high BMD may cause higher vertebral stiffness, and by this mechanism, mechanical stress on the disc may cause repetitive tears in the annulus fibrosus and result in compression and herniation of the disc [14]. As the accumulation of small tears in the disc through these mechanical and vascular pathophysiological pathways occurs over many years, and the structure of the disc is stronger in young adults, high BMD may not result in LDH in young people through these mechanisms. Thus, we conducted this study to evaluate the relationship between BMD and LDH in postmenopausal women.

## Materials and methods

Study design

This study was a retrospective cross-sectional analysis based on an electronic database from a single center.

Patient selection

An analysis of 545 consecutive patients at a state hospital between January 2021 and February 2022 was performed. Inclusion criteria were a diagnosis of low back pain with or without radiculopathy that persisted for at least one-month, postmenopausal women, patients aged 45 to 85 years, and patients who had undergone both a BMD and a lumbar MRI within one month. Patients with malignancy, inflammatory joint disease, vertebral fractures, a history of spinal surgery for any reason, and those using medications that could affect bone metabolism were excluded from the study.

Outcome measures and confounders

Age, weight, height, L1-L4 BMD, L1-L4 T-score, L1-L4 Z-score, femoral neck BMD, femoral neck T-score, and femoral neck Z-score of the patients were collected from the electronic database of the hospital. Patients' weights and heights were measured before BMD measurements using a Soehnle Professional 7830 electronic scale (100g precision) and a standard stadiometer (1mm precision), respectively. Body mass index (BMI) was calculated by dividing the patient's body weight by the square of their height and was expressed as kg/m². BMI was stratified as: 18.5 to 24.9 kg/m², normal; 25.0 to 29.9 kg/m², overweight; 30.0 to 39.9 kg/m², obese; and ≥40 kg/m², morbidly obese, according to the World Health Organization (WHO) definitions.

According to WHO guidelines, a T-score between -1 and -2.5 was diagnosed as osteopenia, and a T-score of -2.5 or less as osteoporosis. Patients with a T-score of -1 or above were included in the normal BMD group. BMD measurements were performed using the dual energy X-ray absorptiometry (DXA) method using the LUNAR DPX densitometer (Madison Corporation, USA). MRI scans were obtained using a 1.5 Tesla machine (Philips, Suzhou, China). LDH was defined as "localized or focal displacement of disc material beyond the limits of the intervertebral disc space," according to the latest recommendations of the North American Spine Society [15]. The term "localized" or "focal" was defined as the extension of the disc material to less than 25% of the circumference of the disc as viewed in the axial plane [11]. The MRI scans (L1 to S1) were assessed by an independent reader who was blinded to the patients' details. This study was approved by the Institutional Review Board (Approval Number: 2022-1/23). A written informed consent was obtained from all individual participants included in the study.

Statistical analysis

Data were included in a database created by the Excel 2007 program by Microsoft (Microsoft Corporation, Redmond, Washington, USA). Statistical analysis was performed using PASW statistics for Windows (version 18, USA). The Shapiro-Wilk test was used to determine whether the continuous variables were normally distributed. Continuous variables that provided the parametric test assumption in comparison between groups were tested with Student's t-test. Categorical variables were compared using the Chi-Square test. A binary logistic regression analysis was performed to adjust for the effects of covariates, including age and BMI. To explore the impact of the number of herniated segments, patients with LDH were further divided into single-level and multi-level LDH groups. An ANOVA test was performed to analyze the relationship between herniated segment numbers and BMD. A p-value of <0.05 was considered significant.

## Results

Characteristics of patients

Five hundred and forty-five postmenopausal women were initially enrolled in the study. After the exclusion of six patients with malignancy, 12 patients with vertebral fractures, two patients with rheumatoid arthritis, nine patients with a history of spinal surgery, one patient using glucocorticoids, and two patients using anti-osteoporotic drugs, 513 patients were included in the final analysis. Four hundred and six patients had at least one level of LDH, and 107 patients had normal MRI findings. The mean age of the patients was 61.3±8.6 years in the LDH group and 58.4±7.8 years in the non-LDH group (p=0.001). The two groups were comparable in terms of height, body weight, and BMI (p=0.392, 0.324, and 0.154, respectively) (Table 1).

**Table 1:** Characteristics of the patients. Notes: Variables are expressed as the mean and standard deviation. A student-T test was used to compare the LDH and non-LDH groups. LDH: Lumbar disc herniation, BMD: Bone mineral density.

Characteristic	LDH	Non-LDH	P-value
Sample size	406 (79.1%)	107 (20.9%)	-
Age, years	61.3±8.6	58.4±7.8	0.001
Height, cm	157.7±6.5	158.2±5.2	0.392
Weight, kg	75.2±12.3	73.8±13.2	0.324
BMI, kg/m²	30.3±5.0	29.5±5.1	0.154
L1-4 BMD	1.1±0.2	1.0±0.1	<0.001
L1-4 T score	-0.6±1.3	-1.2±1.2	<0.001
L1-4 Z score	0.1±1.2	-0.6±1.0	<0.001
Femur neck BMD	0.9±0.1	0.8±0.1	0.004
Femur neck T score	-0.8±1.1	-1.0±0.9	0.023
Femur neck Z score	0.1±0.9	-0.3±0.7	<0.001

Lumbar disc herniation: bone mineral density relationship

The LDH group had higher lumbar and femoral neck BMD, T, and Z scores compared to the non-LDH group (p<0.05) (Table 1). In binary logistic regression analysis, the covariate-adjusted model showed that higher age, higher lumbar L1-L4 BMD, and higher femoral neck BMD were significantly associated with an increased risk of LDH (p<0.05) (Table 2).

**Table 2:** Factors associated with lumbar disc herniation and binary logistic regression analysis results (the covariate-adjusted model). Notes: n=513. Covariates included in the model for statistical analysis are: age, BMI, L1-4 BMD, and Femur neck BMD. BMI: Body mass index, BMD: Bone mineral density.

Variable	Odds ratio (95% CI)	P-value
Age, years (per 1 year increase)	1.08 (1.05-1.12)	<0.001
BMI, kg/m² (per 1 kg/ m² increase)	0.98 (0.93-1.02)	0.332
L1-4 BMD (per 0.1 g/cm² increase)	1.35 (1.11-1.64)	0.003
Femur neck BMD (per 0.1 g/cm² increase)	1.32 (1.01-1.72)	0.040

The relationship between lumbar disc herniation and T-scores

The proportion of patients with a normal L1-L4 T-score was 58.4% in the LDH group and 41.1% in the non-LDH group (p=0.001, OR 95%CI: 2.01 [1.30-3.10]). The proportions of patients with a normal femoral neck T-score were 59.6% and 48.6% in the LDH group and non-LDH group, respectively (p=0.041, OR 95% CI: 1.56 [1.02-2.39]) (Table 3).

**Table 3:** Relationship between LDH and T-scores. Notes: The Chi-Square test was used to compare the LDH and non-LDH groups. LDH: lumbar disc herniation.

	LDH group (n=406)	Non-LDH group (n=107)	Odds ratio (95% CI)	P-value
Normal (≥1) L1-L4 T-score	58.4%	41.1%	2.01 (1.30-3.10)	0.001
Normal (≥1) femur neck T-score	59.6%	48.6%	1.56 (1.02-2.39)	0.041

Analysis of patients according to the number of herniated segments

There was no association between the single and multi-level herniation groups in terms of L1-4 and femoral neck BMD (p=0.760 and 0.435, respectively).

Patients with multi-level herniation had significantly higher rates of obesity (BMI ≥30) compared with single-level herniation (58.0% vs. 47.0%; p=0.031). However, in terms of obesity rates, the LDH group and the non-LDH group were statistically similar (53.9% vs. 54.2%; p=0.961).

## Discussion

While the effect of BMD on disc degeneration and osteoarthritis has been widely observed in previous studies [16-20], the LDH-BMD relationship has been understudied. Previous studies were conducted in a young, healthy adult population collected from another study or in young patients with a small sample size [10,11]. We evaluated postmenopausal women who suffer from low back pain, and our results indicated that, after adjusting for covariates, higher lumbar BMD and higher femoral neck BMD were significantly associated with an increased risk of LDH (p<0.05).

Lumbar end plates are the main anatomical structures that carry loads from bone to disc. Stronger bones mean more load from end plates to intervertebral discs [9]. These anatomical regions also contain small vessels for the supply of the disc. Therefore, the morphology, content, and density of these plates are crucial for the integrity of the discs [21]. Structural changes in the end plates can cause higher pressures on the disc and, thus, herniation of the disc.

Several studies have reported that osteophytes and end plate sclerosis may cause a false increase in DXA measurements [22-24]. Therefore, some recent studies have used quantitative computerized tomography instead of DXA and measured BMD from the lumbar trabecular bone region [10,25]. In the present study, we evaluated both L1-L4 and femoral neck BMD using the DXA technique to better interpret the relationship between LDH and the systemic BMD. Both L1-L4 and femoral neck BMD were significantly associated with LDH (p<0.05).

The incidence of LDH has been shown to increase with age [7,10,11]. In the present study, consistent with previous literature, there was a positive correlation between the age of the patients and LDH (p=0.001).

In a previous study, BMI was significantly higher in patients with ≥2 herniated segments than in healthy adults [10]. In the present study, patients with multi-level herniation had significantly higher rates of obesity (BMI ≥30) compared with single-level herniation (58.0% vs. 47.0%; p=0.031). However, in terms of obesity rates, the LDH group and the non-LDH group were statistically similar (53.9% vs. 54.2%; p=0.961). Despite the findings of the present study suggesting that obesity may be associated with multi-level LDH, we must emphasize that an association is not always causal, and patients may develop obesity because of reduced activity due to their low back pain.

Consistent with previous literature [10], the number of herniated segments was not significantly associated with BMD in our study. Although we did not find any significant differences between subgroups, the observation needs to be further investigated by longitudinal studies. The reason for the lack of statistically significant differences between multi-level LDH and single-level LDH subgroups may be related to the initiation of treatment after the diagnosis of the first LDH in many patients. This may prevent the development of multi-level LDH in these patients. This issue can be better evaluated with longitudinal studies.

LDH is one of the most common causes of low back pain [26]. LDH was present in 79.1% of patients with low back pain in the present study. In a previous study, 66.4% of patients with low back pain had LDH [27]. However, in their study, the population was younger, and the patients included were between 20 and 64 years of age [27].

Differently from previous studies regarding LDH, we selected our study group from postmenopausal women. With the rapid decrease in estrogen levels, menopause both causes rapid loss of bone mass as well as accelerated lumbar disc degeneration [28,29]. As we only included postmenopausal patients, we minimized the effect of estrogen level differences on outcomes. Moreover, we evaluated the femoral neck BMD in addition to the L1-L4 BMD. This prevented a possible misinterpretation of BMD levels due to lumbar osteophytes and end plate sclerosis. However, this study has several limitations. The first limitation was the retrospective cross-sectional evaluation. The second limitation was that some variables were not available, including smoking status and occupational factors. Third, although both the femoral neck and L1-L4 DXA were assessed, quantitative computerized tomography was not used in this study to eliminate possible false increases in the L1-L4 BMD levels.

## Conclusions

Higher lumbar bone mineral density and higher femoral neck bone mineral density were found to be associated with lumber disc herniation in postmenopausal women with low back pain. These results suggest that bone mineral density assessment may be useful in clinical practice to determine which patients are at higher risk of lumbar disc herniation. According to this assessment, preventive methods such as activity modification, exercise programs, and frequent radiological follow-up can be recommended to patients before lumbar disc herniation develops or progresses. Considering the retrospective cross-sectional analysis, further longitudinal and prospective studies are needed to confirm the results of the current study.
